# Nonpolypous Hamartomas of the Gastrointestinal Tract: An Updated Review on Classification, Denominations, and Clinical Management

**DOI:** 10.1155/2022/6983460

**Published:** 2022-05-09

**Authors:** Simona Gurzu, Diana Burlacu, Ioan Jung

**Affiliations:** ^1^Department of Pathology, George Emil Palade University of Medicine, Pharmacy, Sciences and Technology, Targu Mures, Romania; ^2^Research Center of Oncopathology and Transdisciplinary Research (CCOMT), George Emil Palade University of Medicine, Pharmacy, Sciences and Technology, Targu Mures, Romania; ^3^Department of Pathology, Clinical County Emergency Hospital, Targu Mures, Romania

## Abstract

**Purpose:**

To perform the first systematic report about histological subtypes of nonpolypous hamartomas of the gastrointestinal (GI) tract, from esophagus to anal canal.

**Design:**

From over 19,000 studies about hamartomas, most of them published as case series or case presentations, we have selected the most representative ones for the GI tract, excluding polyposis syndromes. To have a whole picture of these hamartomas, all of the data were combined with the personal experience of the authors who are GI pathologists.

**Results:**

The examined articles showed predominance of vascular and combined vascular and mesenchymal hamartomas. Arteriovenous hamartomas or Brunner gland hamartomas are mainly diagnosed in the small intestine, with preponderance for duodenum. Other malformations such cavernous hamartomas are more specific for the colorectal segments, whereas chondromatous hamartomas or those derived from the neural ectoderm were mostly reported in the esophagus. As newly recognized entities were admitted in the last years, misdiagnosis is frequent, and the best therapeutic approach is far to be known.

**Conclusion:**

Even rare, hamartomas of the GI tract need to be differentiated from tumors and familial polyposis syndromes. Knowing their proper denominations and possible complications is valuable for gastroenterologists, pathologists, and surgeons, to be aware in the differential diagnosis.

## 1. Introduction

Hamartomas are rare developmental aberrations that occur in 1 of 3000-4000 live births [1]. The term derives from the Greek word “hamartia” meaning error or failure. Albrecht defined these “tumor-like malformations” in 1904 as being composed by normal cells and tissue components, like the organ in which they occur, which show a disorganized architecture and a predominance of one component [2, 3]. As the diagnosis is frequently difficult, improper nomenclature and misdiagnoses can prolong the diagnosis time, and therapeutic approach requires a multidisciplinary team [4].

In gastrointestinal (GI) tract, hamartomas are uncommon and usually affect children and young adults. They can occur anywhere in the submucosa and rare in the mucosa of the GI segments, from esophagus to the anal canal. At diagnosis, it is necessary to take into account that a GI tract hamartoma can frequently associate other malformations of the GI tract, pancreas, vertebrae, or other organs [1].

The two main groups of GI hamartomas are the hamartomatous familial gastrointestinal polyposis [5] and vascular hamartomas [4, 6–8]. The other uncommon subtypes include mesenchymal [3] and heterotopic hamartomas (e.g., ectopic pancreas or gastric mucosa, gastric duplication, and duplication cysts) [9–11].

The aim of this review is to synthesize the literature data regarding classification, evolution, therapeutic approaches, and incidence of these lesions. Only one previous similar review was published this year (2022) which refers to symptomatic polypoid hamartomas of the jejunum and ileum of 39 adults and 10 children [12]. As most of the gastrointestinal hamartomas reports are limited to single cases or case series, this is the first review addressing the histological subtypes of nonpolypous GI hamartomas, from esophagus to anal canal.

## 2. Methodology

To elaborate this review, a systematic search of the literature was undertaken to identify those representative papers which show classic and particular features of GI hamartomas. It was focused on the whole clinical data, from incidence to therapeutic approaches.

To enrich the above-mentioned aim, we have performed a systematic review of papers indexed in Medline (PubMed) and Web of Science databases. All data available till March 2022 were included. The following search terms were used as combined with hamartoma: “gastrointestinal” “esophagus”, “stomach”, “intestine”, “duodenum”, “jejunum”, “ileum”, “ileocecal”, “diverticul”, “colon”, “cecum”, “rectum”, and “anal canal”. The other searched MeSH terms and text words were “angiodysplasia”, “vascular malformation and gastrointestinal”, “neuromuscular and vascular hamartoma”, “caliber persistent artery”, “Dieulafoy lesion”, “duplication cyst and gastrointestinal”, “tailgut cyst”, “Brunner gland hamartoma”, “hemangioma and gastrointestinal”, and “lymphangioma and gastrointestinal”. Data assessment was conducted independently by two of the three authors (GS, BD, and JI) using predefined terms.

**Figure 1:**
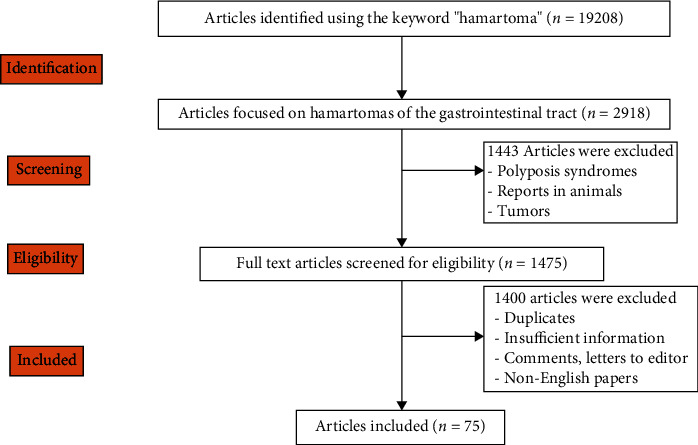
Preferred reported items for systematic reviews and meta-analyses (PRISMA) flow diagram for searching the PubMed and Web of Science databases between 2000 and 2022.

From the research, we have eliminated the hamartomatous familial gastrointestinal polyposis syndromes which include Cowden syndrome, juvenile polyposis, Peutz-Jeghers syndrome, Bannayan-Riley-Ruvalcaba syndrome, hereditary mixed polyposis syndrome, Gorlin syndrome, Birt-Hogg-Dube syndrome, neurofibromatosis type I, and multiple endocrine neoplasia syndrome 2B [5] (Figure 1).

## 3. Vascular Hamartomas

In 1996, the International Society for the Study of Vascular Anomalies (ISSVA) firstly classified vascular aberations into vascular malformations and proliferative vascular lesions (tumors). This classification was updated in 2014 and grouped them in simple and combined vascular malformations [4]. Simple malformations include capillary-, venous-, arteriovenous-, and lymphatic malformations, same as arteriovenous fistula, whereas combination between vessels (more than two vascular malformations in the same lesion) is considered a combined malformation [4].

In GI tract, vascular hamartomas are rather not diagnosed than rare and are mostly arteriovenous type [4, 6]. Denominations such as “caliber-persistence” or “caliber-persistent artery,” “submucosal aneurysm,” “large submucosal arteries,” “angiodysplasia,” “submucosal arteriole malformation,” “vascular malformation,” and “gastric arteriosclerosis” are used in daily practice [6]. In the French literature, the “caliber persistence” is also known as “Dieulafoy's lesion” [6, 13]. Although the 2014 updated ISSVA classification includes hemangiomas and lymphangiomas in the group of tumors, the World Health Organization considers them as malformative lesions of the GI tract [4].

### 3.1. Arteriovenous Hamartomas

They are developed in GI submucosa and consist of proliferation of malformed capillaries and large arterial and venous structures, some of them with sinuous aspect [4, 14]. A characteristic feature is protrusion of the large vessels from submucosa, through muscularis mucosae, in mucosa, predisposing to occult or fulminant and even lethal hemorrhage [6, 15, 16]. Arteriovenous communications and shunting might coexist [4].

First description of Dieulafoy's lesion was provided by Gallard in 1884 who called it as “millier aneurysm of stomach” [6]. The French pathologist Paul Georges Dieulafoy described it in 1897 as “exulceratio simplex” [6, 7]. The term was then changed by Voth in German literature in 1962 and firstly used by Krasznai in English literature in 1968 as “caliber persistence” [15, 16]. Nowadays, it is defined as a small erosion of the GI mucosa, due to a large caliber and persistent submucosal arteriole, and is the cause of 0.5%-14% of upper GI bleeding in adults [13]. In children and young people, this syndrome is rare (only 27 reported cases till 2019) but can be represented by multiple GI erosions and be part of PHACE syndrome (posterior fossa brain malformations, hemangiomas, arterial lesions, cardiac, and eye abnormalities) [13].

In English literature, Moore et al. firstly performed a classification of arteriovenous malformations of the GI tract in 1976, based on angiographic examination [17]. They split them in three groups: type 1: solitary lesions, more frequent in the proximal colon; type 2: large malformations, frequently flat, commonly located in the small intestine; and type 3: punctate angiomas associated or not to hereditary hemorrhagic telangiectasia (Rendu-Osler-Weber syndrome) [17]. Type 1 are acquired lesions of elderly people, whereas types 2 and 3 are rather malformative and affect younger ones [14].

Arteriovenous malformations are more frequent (up to 75%) in the proximal stomach, involving branches of the right gastric artery, followed by the small intestine [6, 13] but are uncommon in the inferior mesenteric artery region [18]. The acquired vascular disorders of the submucosal layer can be differentiated at angiography, from congenital vascular malformations (hamartomas), based on the intense and persistent vein opacification in the venous phase, as result of a rapid venous filling [14].

In some cases, these hamartomas can associate carcinomas that are usually diagnosed in early stage, especially in the stomach [8]. In other cases, congenital malformations such as Meckel's diverticulum may serve as a place of development of an arteriovenous malformation [14]. Treatment consists in vascular embolization but, as this therapeutic approach can induce intestinal ischemia, surgical resection is recommended in selected patients. In children, antiangiogenic prolonged (3 years) therapy with rapamycin or other substances was proved effective to reduce GI bleeding and inhibit angio- and lymphangiogenesis [13].

### 3.2. Venous Hamartomas/Cavernous Hemangiomas

These rare malformations of distal colon and rectum were described in 1839 by Philips and only 351 cases were reported in Medline database till 2021 [19, 20]. In patients with anemia and occult, minimal, or fulminant rectal bleeding, the endoscopy can reveal unspecific lesions, from purple mucosa, submucosal petechiae, or fluid-filled cystic structures, which induce prolonged bleeding, after biopsy, till circumferential thickening of the GI wall and formation of phleboliths in 29-50% of the cases [19, 20]. Differential diagnosis includes hemorrhoids, pneumatosis or melanosis coli, ulcerative colitis, proctitis, and carcinoma [19, 20].

### 3.3. Lymphangiomas

They are composed by dilated lymphatic channels or cysts that are lined by podoplanin (D2-40) or LYVE-1 positive-endothelial cells and predominantly occur in the small intestine [4, 21]. Based on the size of the dilated vessels, lymphangiomas are classified in capillary, cavernous and cystic lymphangiomas [4, 21]. If capillary and cavernous lymphangiomas are mainly developed in the submucosa of the GI tract, cystic lymphangioma is rather a mesenteric lesion developed as result of the mTOR pathway activation, which is therapeutically inhibited by everolimus [21, 22].

### 3.4. Hemolymphangioma

It is a rare variant of lymphangioma, with venous and lymphatic component, which estimated incidence is 1.2 to 2.8 per 1000 newborn infants [21]. Only 8 cases were reported in adults, all of them in the small intestine [21]. Endoscopic or open surgery removal, along with endoscopic sclerotherapy, was used in the reported cases, as the therapy of choice [21].

Lymphangiomatosis, which is a multisystem presence of lymphangiomas, can involve the GI tract along with other organs such as the liver, spleen, or kidney [23]. GI lymphangiomatosis might associate occult anemia, spontaneous bleeding, and abdominal pain and can be complicated by protein-losing enteropathy [23]. In such cases, besides removal of the lesion, when necessary, systemic therapy with rapamycin or thalidomide is recommended [23].

### 3.5. Multifocal Lymphangioendotheliomatosis

It is a cutaneous-visceral lesion consisting on association of GI vascular hamartomas, which are characterized by distinct endothelial proliferation marked immunohistochemically by the lymphatic vessel endothelial LYVE-1 or hyaluronan receptor 1, with diffuse congenital vascular lesions of the skin, lung, spleen, choroid plexus, bones, etc. [24–26]. It might be complicated with thrombocytopenia refractory to blood cell transfusions and life-threatening bleeding, with a subsequent mortality rate of 65% in newborns [24, 27]. As this is a newly recognized entity, which was firstly described by North et al., there is no consensus on the proper therapy [25, 28]. The rapamycin inhibitors such as sirolimus, combination of vincristine and prednisolone, and association of aminocaproic acid, octreotide drip, and corticosteroids were used in the cases reported in literature [25–28]. In refractory cases, the antiangiogenic therapy with bevacizumab was suggested to be used [27].

## 4. Combined Vascular and Mesenchymal Hamartomas

### 4.1. Neuromuscular and Vascular Hamartoma

Neuromuscular and vascular hamartoma (NMVH), which is also known as fibrovascular hamartoma, represents a variant of vascular hamartomas in which the proliferated vessels are relatively small and mesenchymal components are associated [29]. Macroscopically, NMVH is a localized lesion of the submucosa of the small intestine, usually hemorrhagic, which is covered by normal or ulcerated mucosa and can protrude in the intestinal lumen [29, 30]. Uni- and multifocal thickening of the submucosa, with stenosis of the intestinal lumen, were also described [30, 31]. Small intestine contrast-enhanced ultrasonography is considered as the optimal imagistic method for diagnosis of NMVH [29].

Under light microscope, the overlying mucosa is intact or ulcerated, the muscularis mucosae appears disorganized, and submucosa is enlarged and encompasses fascicles of smooth muscle derived from the muscularis mucosae admixed, in a collagenous stroma, with hemangiomatous and lymphatic vessels, neural fibers, and ganglion-like cells with or without adipose tissue or other mesenchymal structures [30–33]. Amyloidosis and presence of inflammatory cells or lymphoid follicles at the upper edge of NMVH and focal duplication of the muscularis propria were also described [29, 31]. The proliferated vessels are small, with thin walls, or mature and positive to CD31/CD105 and smooth muscle actin (SMA) [34] and can show vasculitis, venopathic changes (“vessel-in-vessel” appearance of the veins, myointimal hump, concentric venous myohypertrophy, obliterative venopathy, etc.), or arteriopathies (concentric arterial medial hypertrophy, elastotic degeneration and crumpling of arterial elastic lamina, etc.) [31].

Fernando and McGovern described first two cases of NMVH, one in ileum and the other one in jejunum of two females (30 and 36 years) in 1982 [30]. Other 27 cases of the small intestine, located in jejunum or ileum of patients aged between 12 and 76 years, with a median age of 53.7 years and a male to female ratio of 1 : 1.5, were described till 2021 [29–33, 35]. Except the small intestine, NMVH can also involve the cecum, with two reported cases, one in a 76-year-old female [36] and one in a 13-year-old boy [37]. Sasaki et al. described one in the appendix of a 60-year-old male in 2020 [32]. One case of NMVH that was developed in a Meckel diverticulum was also reported in 2009 [38].

The clinical symptoms are nonspecific and consist of intermittent abdominal pain, vomiting, loss of weight and appetite, obstructive symptoms, diarrhea, occult hemorrhages, anemia, etc. [31–33]. In cecum, they can induce intussusception [37] and in appendix they can mimic an appendicitis [32].

Differential diagnosis of muscularization of the submucosa includes “burn-out phase” of Crohn's disease, ischemic colitis with hyperplasia of muscularis mucosae, radiation enteritis, cryptogenic multifocal ulcerous stenosing enteritis (CMUSE), and other chronic inflammatory bowel diseases (IBDs) [30–32, 35, 39]. In chronic inflammatory lesions, NMVH-like aspect can be induced by the mesenchymal morphologic response to inflammation [31] which is also known as type 2 epithelial to mesenchymal transition/mesenchymal metaplasia/neomuscularization responsible by tissue repair/fibrogenesis [31, 40, 41]. A similar lesion can be induced by consumption of nonsteroidal anti-inflammatory drugs (NSAIDs) and, because it is like a diaphragm-like annular stricture of the intestine, it was called “diaphragm disease” [29, 31]. In hamartomas with prominent neural proliferation, neurinomatous hyperplastic lesions such as paragangliomas, ganglioneuromas, glomus tumors, and neurofibromatosis need to be excluded [30].

### 4.2. Neuromesenchymal and Vascular Hamartoma

**Figure 2:**
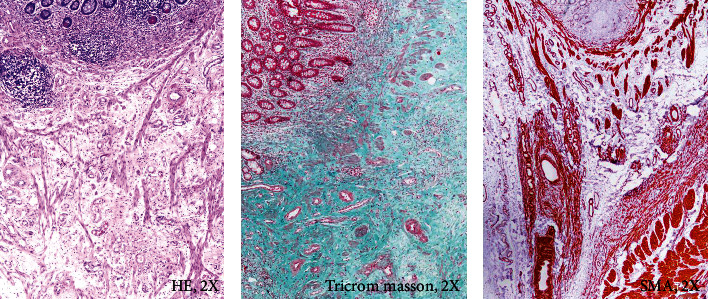
Microscopic and immunohistochemical features of neuromuscular and vascular hamartoma: enlarged submucosa with proliferation of small vessels marked by smooth muscle actin (SMA), embedded in a collagenous stroma.

It is similar with NMVH but the mesenchymal components like adipose tissue and fibrous tissue are better represented [33]. In one of our cases, a similar lesion was found in the cecum of a 79-year-old female, without personal history of IBD or other significant diseases. This patient presented with obstructive symptoms and endoscopy was not successful, due to obstruction of the lumen. The abdominal contrasting computed tomography (CT) revealed the suspicion of an unusual cecal lipoma in which the contrast substance was absorbed in the upper edge of the lesion. Histopathological diagnosis revealed a well-defined lipomatous lesion covered by normal mucosa with submucosal proliferation of small podoplanin-positive lymphatic vessels, along with arterial and venous structures marked by CD31 and CD34, some of them enlarged or protruding into the mucosa. They were embedded in a collagenous stroma and in submucosa, muscularis mucosa was disorganized, and smooth muscle fiber bundles were scattered between the proliferated vessels (Figure 2). After surgical removal of hamartomatous lesion, no recurrences or other complications were reported after 13-month follow-up.

## 5. Mesenchymal Hamartomas

### 5.1. Mucosal Schwann Cell Hamartoma

It is an uncommon sessile lesion of the GI tract mucosa characterized by ill-defined proliferation of Schwann cells in the lamina propria, without whorls, palisading, fasciculation, or necrosis [34, 42]. These S100-positive elongated spindle cells have tapering small nuclei and well-defined eosinophilic cytoplasm and are usually negative for neurofilament protein (NFP) [34, 43, 44]. In the past, these lesions were known as neuroma [45].

Gibson and Hornick described the first 26 cases of mucosal Schwann cell hamartomas of the colon presented as mucosal polyps [43]. Between 2008 and 2021, other 60 cases were reported. The 86 cases of the appendix (*n* = 3), proximal colon (*n* 16), distal colon (*n* = 57), and rectum (*n* = 10) occurred in males and females with a median age of 60.2 years [43, 44]. Although Schwann cell hamartoma was considered as exclusively occurring in the colon, a series of 6 cases of gastroesophageal junction was published in 2020 by Li et al. [45].

As mucosal Schwann cell hamartomas can be multiple (4/86 cases), differential diagnosis includes von Recklinghausen's (type 1) neurofibromatosis, in which the mucosal crypts are not affected, and multiple neuromas from patients with multiple endocrine neoplasia type 2B (MEN 2B) [42–44]. As opposite, schwannoma is well circumscribed but not capsulated, is surrounded by a lymphoid peripheral cuff and is usually a solitary tumor [42, 43]. Schwannoma is more frequent in the distal colon followed by stomach and gastroesophageal junction [42–44]. Solitary ganglioneuroma, neurofibroma (ganglion cells are positive for CD34), leiomyoma (positive for smooth muscle actin), gastrointestinal stromal tumor (GIST), and perineuroma (formerly known as “benign fibroblastic polyp”) should also be excluded [34, 41, 45].

### 5.2. Neural Ectoderm-Derived Hamartomas

Systematized epidermal nevi of the esophagus are considered hamartomatous lesions originated from the neural ectoderm. This multisystemic syndrome mostly involves the brain, head and neck area, arms, and trunk. Oyesanya et al. reported the first case of verrucous epidermal naevi of the esophagus [46].

### 5.3. Rhabdomyomatous Mesenchymal Hamartoma

Hendrick et al. first described this “striated muscle hamartoma” in 1986 [47]. It is also known as “congenital midline hamartoma,” “hamartoma of cutaneous adnexa and mesenchyme,” and “skin tag hamartoma” [48, 49]. The commonest location is the deep dermis and the subcutaneous adipose tissue of the head and neck area of children (34 out of 46 cases reported up to 2016 and 88% of those published till 2019) but can also affect the jaw and rarely the tongue, orbit, back, appendages of the sacrum, digits, and even Eustachian tube [47–49]. Only four cases were reported in the perianal region [48, 49]. Rhabdomyomatous mesenchymal hamartomas are diagnosed as single or multiple papules or pedunculated or sessile polypoid lesions and are microscopically composed by randomly arranged disorganized desmin or myogenin-positive mature skeletal muscle fibers and myofibroblasts [48, 49]. Cleft lip/palate is commonly associated [49]. Differential diagnosis includes smooth muscle hamartoma, fetal rhabdomyoma/sarcoma, nevus lipomatous superficialis, infantile myofibromatosis, benign triton tumor (showing CTNNB1 mutation), and Langerhans-cell histiocytosis [47, 49]. In some cases, rhabdomyomatous mesenchymal hamartoma was described as part of the amnion band syndrome or Delleman (oculocerebrocutaneous) syndrome, which is characterized by absence of corpus callosum associated with colobomas, orbital cysts, cerebral cysts, skin tags, etc. [49].

### 5.4. Chondroma-Like Hamartomas

Chondromatous hamartoma is an uncommon intramural lesion of the esophagus composed by hyaline cartilage, spindle cells, and glandular structures [50]. It represents <7% of all benign polypoid lesions of the esophagus and are mainly located between muscle and mucosal layer and can be endoscopically resected [3, 50]. Differential diagnosis should firstly include low-grade chondrosarcoma, choristoma, and teratoma [50, 51].

Osteochondromatous hamartoma is a variant of chondromatous hamartoma in which cartilage proliferation is accompanied by bone, fat, and fibrous tissue [3]. Dysphagia, dry cough, vocal cord paralysis, vomiting, and infrequent weight loss can accompany these intramural lesions [50].

Chondroid hamartoma is composed by chondroid, adipose, and fibrous connective tissue [51].

Chondromesenchymal, angiomatous, lipomatous, and leiomyomatous hamartomas are other described variants [51]. Only 19 cases of esophageal hamartomas were reported to date, from which 8 involved the upper segment [50, 51]. Leiomyomatosis-like lymphangioleiomyomatosis was reported in three case reports, as a manifestation of tuberous sclerosis in colon and ileocecal segments. The epithelioid component can express melanoma markers such HMB-45 but is negative for C-kit, CD34, and DOG-1, which are characteristic for GISTs [52].

## 6. Heterotopic Hamartomas

### 6.1. Ectopic Pancreas

It is defined as abnormal presence of pancreatic tissue in anatomical areas which do not have continuity with the pancreas [9, 53]. It can be a solid or cystic lesion which mostly involves the stomach and small intestine but can be also developed in a Meckel's diverticulum, cystic duplication or other GI hamartomas [9, 53]. In 1909, von Heinrich classified the ectopic pancreas in two main groups, the classification being still available. It is about identification of ectopic tissue comprising ducts, acini, and Langerhans islands (type 1), presence of ducts and acini (type 2), or cystically dilated ducts only, without acini or endocrine component (Heinrich's type 3) [9, 54]. The main complications are hamartomatous chronic pancreatitis with pseudocyst formation, gastric outlet obstruction, and malignization [55].

### 6.2. Myoepithelial/Myoglandular Hamartoma

This hamartoma, which is also known as adenomyosis or ademyomata, is a variant of Heinrich's type 2 ectopic pancreas which appears as a submucosal mass of the GI tract [11, 55–58]. As it is especially located in the gastric antrum (85%) and pylorus (15%), Magnus-Alsleben called it in 1903 “adenoma of the pylorus” [56] being later called adenomyoma [57]. Only 58 cases were reported till 2019 in the stomach and 7 cases in the ampulla of Vater [11, 57, 59]. Under microscope, it is composed by smooth muscle bands admixed with glandular structures, some of them cystically dilated, Brunner or pyloric glands, and trypsin-positive pancreatic acini [11, 57]. It can coexist with other malformations such annular pancreas, gastric duplication, and duodenal adenomas [57]. In newborns, it can mimic the infantile hypertrophic pyloric stenosis [58]. Endoscopic submucosal resection can be the therapy of choice and the malignization rate is <2% [57].

### 6.3. Brunner's Gland Hamartoma

While Brunner described in 1688 the glands with the same name, Cruveilhier described first case of Brunner gland hamartoma in 1835 [60]. Then, the described lesion was separated in three entities: diffuse hyperplasia, localized hyperplasia, and hamartoma or adenoma which was recently denominated as benign glandular hyperplasia [10, 60–63]. It is about nodular or pedunculated uncommon submucosal masses of the duodenum diagnosed in adults [10, 60, 61]. About 70% of the cases involved the pylorus and duodenal bulb [64]. Only one pediatric pyloric localization was reported in literature [62, 63].

According to the Armed Forces Institute of Pathology, solitary or multiple masses below 5 mm in size, composed by excessive Brunner glands separated by fibrous septa, is indicated to be called “hyperplasia” [61, 64]. The term “hamartoma” is reserved to nonencapsulated solitary and larger than 5 mm, even giant masses, composed by sheets of Brunner glands along with acini and ducts, some being dilated, mesenchymal (smooth muscle bundles and adipose tissue), and lymphoid tissue, which are infrequently admixed with heterotopic pancreas [10, 61, 63]. Differentiation from an adenoma (Brunneroma) is not clearly defined in literature, and the two lesions are usually considered similar and were reported in patients between 24 and 76 years [59, 61, 63]. As incidence, 5-10% of duodenal benign lesions are thought to be Brunner's gland hamartomas [63].

Similar to other GI tract hamartomas, patients can be asymptomatic or present nonspecific symptoms such abdominal pain, nausea, bilious vomiting, anemia, and intussusception [10, 61–63]. Treatment consists of endoscopic mucosal resection or surgical excision [10, 60], respectively, longitudinal pyloromyotomy with transverse pyloroplasty [62]. The main complications are intussusception and ileus. Although it was long-time known that malignization is not a complication of Brunner gland hyperplasia/hamartoma, recent data showed dysplasia in 2.1% and associated Brunner glands adenocarcinoma in 0.3% of cases [60, 64, 65].

The Brunner gland hyperplasia needs to be differentiated by polyposis syndromes, pancreatic heterotopia, pyloric/duodenal duplication cysts, nodular lymphoid hyperplasia or duodenitis, and tumors such as neuroendocrine neoplasm, lipoma, and leiomyoma [60, 62, 64].

### 6.4. Heterotopic Gastric Mucosa

It is an uncommon hamartoma of the cervical esophagus which is discovered incidentally in 1-10% of adults during screening endoscopies. It is predominantly composed by funding-gland type (24%) followed by cardiac-gland type mucosa (15%), but most of the cases (61%) include both types [66]. Other segments of the GI tract, such as duodenum, jejunum, ileum, or Meckel's diverticulum, can be infrequently involved. Although endoscopic resection is indicated, malignization was not reported.

## 7. Other Cystic Hamartomas

### 7.1. Duplication Cyst

The estimated incidence of GI tract duplications is 1 : 4500 live births [1]. Although 60% of the subepithelial lesions of the GI tract is supposed to be duplications [67], few cases can be found in English literature [68]. These enteric cysts are especially found in the ileum (33%), esophagus (20%), jejunum (10%), colon (13%), and stomach (2-9%) [1]. The appendix and duodenum are infrequently involved [1, 68–70]. A number of 50 cases were also reported as anal canal duplications [71].

First, jejunal duplication cyst was described in 1950 by Oyama [69] and, as it shares the mesenteric vascularization [67], was called “chyliferous cyst of the mesentery” [69]. Other 30 jejunal duplication cysts were reported till 2016 [9]. In the stomach, the lesion is also known as “double stomach” [68]. Distal grater curvature is mostly involved being followed by the posterior wall, the lesser curvature, the anterior wall, and pylorus [1, 68].

The patients can be asymptomatic, but if they are diagnosed in the first year of life or early childhood, mostly before the age of 12, abdominal pain, vomiting, weight loss, and even a palpable abdominal mass can be revealed [1, 68]. In adults, iron deficiency anemia, bleeding episodes, abdominal pain, nausea, vomiting, pancreatitis, and cholestasis might be the symptoms [68, 70].

These tubulars or spheric malformative structures are attached by the adjacent GI segment and can communicate with the GI lumen or with the appendix or major duodenal papilla [9, 67, 70]. However, communication is not a condition for diagnosis and is not present in up to 80% of the cases [1, 9, 67]. To be diagnosed as a duplication cyst, the presence of a separate cyst-lining mucosa but a common muscular wall with the GI segment sharing a common blood supply is necessary to be proved [1]. Under microscope, the gastric or intestinal mucosa-lined cyst is surrounded by a well-defined smooth muscle coat, without a cleavage plane between the cyst and muscularis propria of the involved GI segment, making it contiguous with the GI wall [1, 9, 68]. In the anal canal duplication, the cyst is lined by squamous or transitional epithelium [71].

Inside the cyst, clear fluid is usually found, but bile and enteroliths were observed in duodenal cysts [70, 72]. The cystic wall might also incorporate an ectopic pancreas [9]. Depending on the localization, diameter, associated complications, and the experience of the surgical team, therapy consists of endoscopic removal or marsupialization, excision of the common wall, bypass, or surgical removal of the affected GI segment through laparotomy or laparoscopic methods [1, 9, 68, 70].

In some cases, they mimicked a neoplasm such as GIST or a neuroendocrine tumor [68]. In 11 cases reported in literature till 2018, gastric duplication cyst was reported as a premalignant lesion which predisposed to further development of carcinomas, especially adenocarcinomas or squamous cell carcinomas, but neuroendocrine tumors can also be developed [1]. In such cases, elevated serum levels of carcinoembryonic antigen (CEA) or CA 19-9 can be found [1]. Although only one case of malignization a duplication of the anal canal was reported [71], the risk of malignization does not depend on the localization [70]. Elevated CEA can be found in patients with duplication cysts before malignization and can serve for identification of associated carcinomas in early stages [1].

### 7.2. Tailgut Cyst or Retrorectal Cystic Hamartoma

It is a duplication cyst located in the retrorectal-presacral space [73, 74]. It derives from a postnatal remnant of the hindgut and can show enteric or neuroenteric components. It is differentiated by the rectal cystic duplication by the absence of the smooth muscle coats. The male to female ratio is 1 : 5. The lesion varies from asymptomatic cyst to cystic structures which prolapses through the anal canal and can mimic hemorrhoids or dermoid cysts [73]. As development of neuroendocrine tumors, adenocarcinomas, and transitional or squamous cell carcinoma was reported in 24 cases [73, 74], the surgically resected specimens need to be attentively evaluated.

## 8. Summary and Future Perspectives

In GI tract, nonpolypous hamartomas proved to be more frequently incidentally diagnosed. Although rarely reported, their incidence seems to not be low, and more attention need to be paid to these mimickers. In some cases, neoplasms might be missed, and fatal hemorrhage or intestinal ileus can be the first symptoms, especially for hamartomas of the small intestine. In other cases, they can be premalignant lesions which evolve to transformation in carcinomas or neuroendocrine tumors. Large cohorts need to be examined to check the real incidence of nonpolypous GI hamartomas, at endoscopy or autopsy. As CEA level might be elevated in serum of patients with GI duplication cysts, this serum marker should not be used as an indicator of presence of a malignant tumor but should raise concerns about presence of a malformative lesion with risk for malignant transformation.

## Data Availability

The literature data used to support the findings of this study are available from the corresponding author upon request. This is a systematic review of literature.
